# (Ti,Al)O_2_ Whiskers Grown during Glow Discharge Nitriding of Ti-6Al-7Nb Alloy

**DOI:** 10.3390/ma14102658

**Published:** 2021-05-19

**Authors:** Krzysztof Szymkiewicz, Jerzy Morgiel, Łukasz Maj, Małgorzata Pomorska

**Affiliations:** Institute of Metallurgy and Materials Science, Polish Academy of Sciences, 25 Reymonta st., 30-059 Kraków, Poland; j.morgiel@imim.pl (J.M.); l.maj@imim.pl (Ł.M.); m.pomorska@imim.pl (M.P.)

**Keywords:** (Ti,Al)O_2_ whiskers, plasma nitriding, Ti6Al7Nb alloy, TEM

## Abstract

Plasma nitriding of titanium alloys is capable of effective surface hardening at temperatures significantly lower than gas nitriding, but at a cost of much stronger surface roughening. Especially interesting are treatments performed at the lower end of the temperature window used in such cases, as they are least damaging to highly polished parts. Therefore identifying the most characteristic defects is of high importance. The present work was aimed at identifying the nature of pin-point bumps formed at the glow discharged plasma nitrided Ti-6Al-7Nb alloy using plan-view scanning and cross-section transmission electron microscopy methods. It helped to establish that these main surface defects developed at the treated surface are (Ti,Al)O_2_ nano-whiskers of diameter from 20 nm to 40 nm, and length up to several hundreds of nanometers. The performed investigation confirmed that the surface imperfection introduced by plasma nitriding at the specified range should be of minor consequences to the mechanical properties of the treated material.

## 1. Introduction

A glow discharge plasma nitriding (PN) of parts made of Ti-6Al-4V and Ti-6Al-7Nb alloys is gaining an upper hand over gas nitriding (GN), as it allows the increase of surface hardness without compromising a high strength of the core material [1]. This is because the former process could be performed at much lower temperatures than the latter one, i.e., the PN secures formation of a continuous and slightly porous TiN surface layer even after processing duration of eight hours carried out at 600–850 °C [2], while achieving the comparable results with the GN of the same duration must be performed at 800–1050 °C [3]. A problem in the case of PN treatment is that it causes a surface roughening, increasing with the treatment temperature [4,5]. Therefore, preserving an original high surface finish of the parts subjected later to the GN is possible only for the treatments performed at the lower end of the temperature range proper for them.

The plan-view observations of the surface of the PN Ti-6Al-4V alloy with the scanning electron microscopy (SEM) method showed that it bears both nodular [6,7] as well as much finer point-like features [7]. It is characteristic for the PN process that the nodules are the coarser, the higher the nitriding temperature, while the bright spots representing point surface defects remain practically the same. The cross-section scanning and transmission electron microscopy (SEM/TEM) investigations not only confirmed the presence of a surface undulation representing the nodular features, but also proved that they increase with the PN temperature both for the Ti-6Al-4V [8] and Ti-6Al-7Nb [2,9] alloys. However, none of the images published so far gave any indication of the nature of the point-like objects observed with the SEM method.

Therefore, the present work was focused on the identifying the point-like defects, being a dominating defect in otherwise relatively smooth surface of the PN Ti-6Al-7Nb treated at relatively low temperature of 620 °C. In this experiment, Ti-6Al-7Nb alloy was chosen, as in many medical applications the Ti-6Al-4V containing a carcinogenic vanadium is already unacceptable. The surface morphology was characterized with the SEM, while cross-sectional microstructure investigations were performed with the TEM methods.

## 2. Experiments

The ~3 mm disc was cut from a rod of the Ti-6Al-7Nb alloy (Al—5.94 wt.%, Nb—7.1 wt.%, Ta—0.05 wt.%, C < 0.005 wt.%, Fe < 0.18 wt.%, N < 0.006 wt.%, O < 0.153 wt.%, H < 0.0012 wt.% H, Ti—balance) rod (*φ* ~ 16 mm) purchased from the Bibus Metals Company (Dąbrowa, Poland). Next, this was ground with sandpapers and polished with fine silica suspension till the R_a_ roughness was <0.05 μm. Finally, all the surfaces were washed with de-ionized water, rinsed with ethanol and dried with hot air.

The PN treatment was performed at the cathode potential of 200 V, provided by a direct current (DC) power supply unit. This was done in a vacuum chamber, which at first was pumped down to 10^−3^ Pa, and subsequently the pure nitrogen (5N) was let in up to ~10^5^ Pa working pressure. Next, the samples were heated with a resistive table up to 620 °C. Afterwards, the glow discharge process was started and maintained for 6 h. Finally, the flow of nitrogen was cut off to stop discharge, the resistive heating was switched off, and the system was cooled down with a chamber. The scheme presenting the sample placing in the chamber is described elsewhere [2].

The surface morphology was investigated with the plan-view observations using a ThermoFisher (Waltham, MA, USA) Scios 2 Dual Beam microscope. The effect of charging accompanying the observations of non-conducting materials was eliminated by its evaporation with carbon. The microstructure characterization of the near surface areas was performed on the lamellas of ~7 μm × 15 μm × 100 μm, presenting the nitrided affected zone in the cross-section, with FEI (Hillsboro, OR, USA) Tecnai G2 SuperTWIN FEG 200 kV transmission electron microscope (TEM) equipped with integrated scanning attachment (STEM) and EDAX detector with Ultra-Thin Window (UTW).

## 3. Results

**Figure 1:**
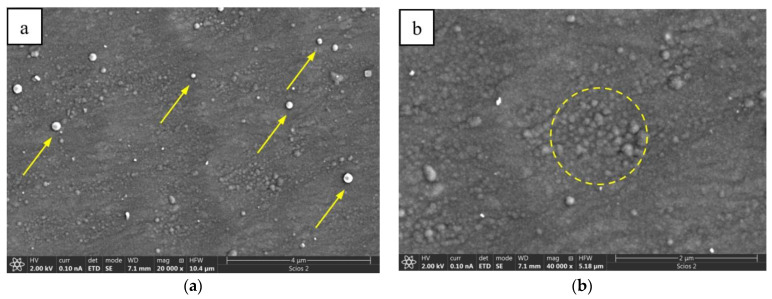
SEM/SE images of PN and carbon coated Ti-6Al-7Nb surface presenting: (**a**) pin-like defects (pointed with arrows) and (**b**) colonies of nodular objects (encircled with dashed line).

The plan-view SEM observations of the PN and carbon coated Ti-6Al-7Nb alloy showed that its surface bears both colonies of the nodular features (encircled with a dashed line) and a number of separate pin-like objects (pointed with arrows) of stronger contrast caused by their higher height (Figure 1). Their density was not higher than 20 pcs per 100 μm^2^. The nodular defects are up to 0.5 μm in diameter and their shape corresponds to that published for the PN Ti-6Al-4V (even as their size is usually much larger probably due to differences in treatment temperature) [6].

**Figure 2:**
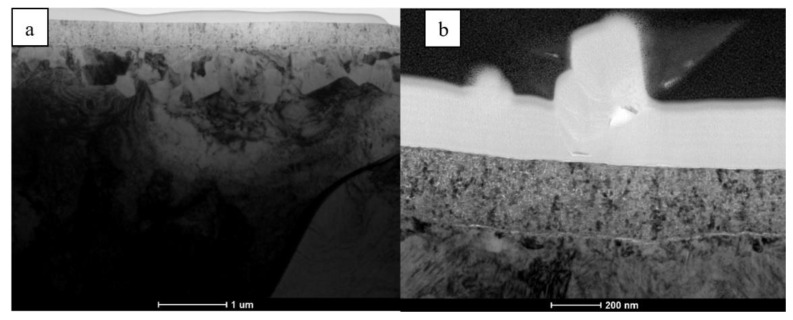
TEM/BF images of cross-section microstructure of TiN layer formed on PN and carbon coated Ti-6Al-7Nb alloy showing: (**a**) overview of smooth surface and (**b**) fragments of nano-whiskers.

The cross-section TEM investigations helped to establish that the carbon deposited on a slightly porous TiN surface layer strongly magnifies all of the surface defects (Figure 2). It is especially misleading in the case of longer bent or even knotted nano-whiskers sticking out from relatively flat surface. Any other defects are also covered by the carbon layer, but their lower height causes a much lesser contrast as compared with that observed for the nano-whiskers.

**Figure 3:**
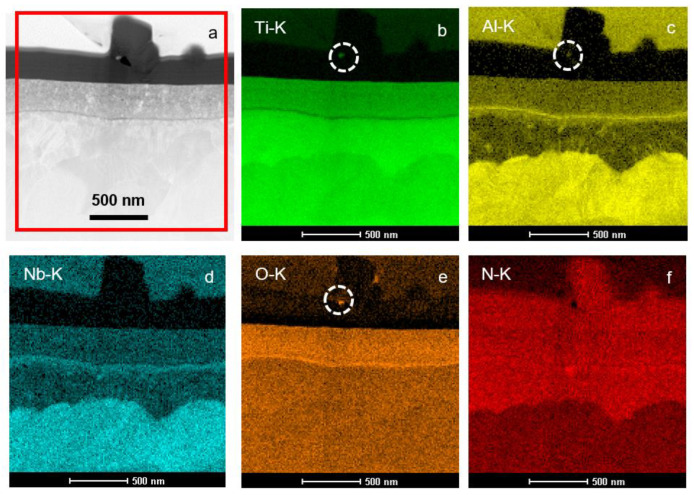
STEM/HAADF image of cross-section microstructure of surface TiN layer with fragments of twisted nano-whisker over it formed on PN and C coated Ti-6Al-7Nb alloy (**a**) as well as accompanying maps presenting distribution of Ti, Al, Nb, N and O (**b**–**f**) elements.

The microstructure characterization of the near-surface areas with the scanning transmission electron microscopy (STEM) using a high angle annular dark field (HAADF) detector indicated the presence of nano-porosity within the upper TiN layer, but pointed toward the possible presence of the string of oxides at its base (identified by a line of darker contrast representing low Z elements) (Figure 3). The maps acquired with the Energy dispersive X-ray spectroscopy EDS system and showing a distribution of the Ti, Al, Nb, N and O elements helped to identify them as aluminium oxide and, to a lesser extent, niobium oxide, i.e., as the remnants of the native oxides formed at the alloy surface in-between the polishing and cleaning process, and the PN treatment. The signal from the whisker fragments turns out to be very weak due to its small dimensions and, in consequence, a small volume was subjected to ionization with the electron beam. However, it clearly showed that these are built predominantly from oxygen, titanium and to much lesser extent with aluminium (see encircled areas on respective EDS elemental maps in Figure 3).

**Figure 4:**
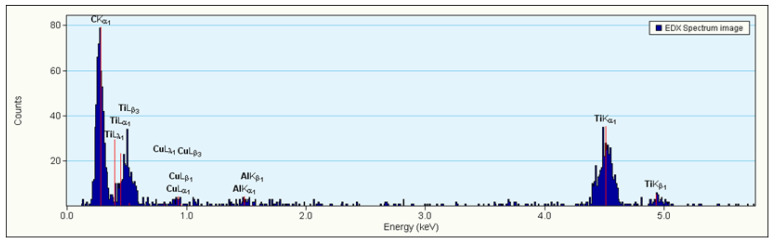
EDS spectra acquired from the fragment of the whisker sticking-out from the surface TiN layer grown on PN Ti-6Al-7Nb alloy.

Even a semi-quantitative assessment of the whisker chemical composition from the elemental maps might be misleading, as in each case the signal level is set against the phase with a maximum content of the chosen element (that is why the content of titanium may seem to be smaller than that of oxygen). However, the EDS spectrum acquired from it with a longer acquisition time clearly documents that the titanium is a dominating element of the whisker, while the aluminium is a lesser addition (with practically no niobium). The standard-less quantification of the metallic elements showed their content at the 20:1 ratio, i.e., ~5 at.% Al against 95 at.% Ti with the oxygen excluded from the estimate due to strong overlap of the O_Kα with Ti_Lα lines (Figure 4).

## 4. Discussion and Summary

The (Ti,Al)O_2_ nano-whiskers documented at the surface of the PN Ti-6Al-7Nb alloy might have formed either from the start of this treatment or may have been nucleated afterwards, i.e., during the first stages of the cooling. The former growth path is to some extent supported by the fact that the analyzed fragments carried aluminium admixture, which source is located below the nitride layer. At that stage it can also draw oxygen from sputtered native oxides. However, it would be also exposed to the sputtering, making this doubtful. The other possibility could be realized after switching off the nitriding system with the residual oxygen, which is present in every vacuum system and capable of reducing the titanium nitride to titanium oxide at this temperature range [10,11]. Therefore, from these two possibilities, the second one seems to be definitely more probable.

It should be noted that titanium and oxygen show a tendency to form rutile whiskers through preferential growth toward [001] direction, even if they grow in wet (hydrolysis of titanium) processes and are relatively thick (above nanometer range) [12]. The presently grown forms were characterized by a much smaller diameter, from 20 to 40 nm, but they stuck above the surface up to 200 nm. In any case, it is their location over the otherwise relatively flat surface which makes them visible, especially in the case of using the SEM observations in secondary electron (SEM/SE) modes. These nano-whiskers definitely form a separate group of defects at the surface of the PN Ti-6Al-7Nb and Ti-6Al-4V alloys, but due to a very small size they should not affect the wear resistance of this material in any significant way.

Identification of the nature of the pin-like surface defects as the (Ti,Al)O_2_ nano-whiskers means that the titanium alloys PN at the lower temperature range, proper for this treatment, may have a better surface quality than that assumed through SEM observations.

## Data Availability

The data presented in this study are available at the Corresponding author.
